# An IFN-γ-related signature predicts prognosis and immunotherapy response in bladder cancer: Results from real-world cohorts

**DOI:** 10.3389/fgene.2022.1100317

**Published:** 2023-01-04

**Authors:** Hao Deng, Dingshan Deng, Tiezheng Qi, Zhi Liu, Longxiang Wu, Junbin Yuan

**Affiliations:** ^1^ Department of Urology, Xiangya Hospital, Central South University, Changsha, China; ^2^ National Clinical Research Center for Geriatric Disorders, Xiangya Hospital, Central South University, Changsha, China; ^3^ Xiangya School of Medicine, Central South University, Changsha, China

**Keywords:** bladder cancer, immunotherapy, IFN-γ, prognosis, real-world cohort study

## Abstract

Bladder cancer (BLCA) is featured with high incidence and mortality. Whether the IFN-γ signaling could be used as an immunotherapy determinant for BLCA has not been fully confirmed. In this study, the transcriptome data and clinical information of BLCA samples were collected from The Cancer Genome Atlas (TCGA). Besides, four immunotherapy cohorts including IMvigor210 cohort, Gide cohort, Van Allen cohort, and Lauss cohort were collected. The Xiangya real-world cohort was used for independent validation. An IFN-γ-related signature was developed and validated in BLCA for predicting prognosis, mutation, tumor microenvironment status, and immunotherapy response. This is the first study focusing on the comprehensive evaluation of predictive values on the IFN-γ-related signature in BLCA. The potential clinical application of the IFN-γ-related signature was expected to be further validated with more prospective clinical cohorts.

## Introduction

Bladder cancer (BLCA) is recognized as one of the most common and heterogeneous urinary carcinomas worldwide (Antoni et al., 2017; Sung et al., 2021). Clinical data ceaselessly confirmed a high incidence and mortality in BLCA patients (Martinez Rodriguez et al., 2017). Thus, for decades, urologists have explored the mysteries of bladder cancer in hope of getting optimal solutions for precision BLCA treatments. Despite surgeries, radiotherapy, neoadjuvant or adjuvant chemotherapy and targeted therapy, BLCA patients still suffer (Antoni et al., 2017; Lenis et al., 2020). Most suffering patients are not sensitive to the current and mainstream treatment methods according to poor clinical outcomes (Lenis et al., 2020; Patel et al., 2020). Therefore, inventing new medical tools and treatment modalities for BLCA patients are urgently needed.

Cancer immunotherapy is a relatively burgeoning section in the field of cancer treatment, providing opportunities for alleviating, even curing BLCA patients (Pettenati and Ingersoll, 2018; Lenis et al., 2020). The immune checkpoint blockade (ICB), considered as the main direction of immunotherapy development, has been observed effective survival benefits in solid cancers, including BLCA (Rosenberg et al., 2016). Further from the cellular and molecular level, the ICB response rate mainly depends on the BLCA tumor microenvironment (TME) (Petitprez et al., 2020; Cao et al., 2021; Marin-Acevedo et al., 2021). TME is chiefly composed of cancer cells and immune cells, with other cell subsets and extracellular matrix as well (Hinshaw and Shevde, 2019). However, a malignant BLCA TME generally leads ICB moving towards failure. Potential mechanisms influencing ICB could be including the exhaustion and senescence of CD8 T cells in TME (Lian et al., 2020; Zhang et al., 2021); the huge secretion immunosuppressive factors (Metelli et al., 2018; Lecker et al., 2021). Besides, high tumor mutation burden (TMB), which could represent a high level of neoantigen, is a prominent characteristic of BLCA indicating a potential immunogenic microenvironment (Kandoth et al., 2013). Even though the response of patients with high TMB to ICB is considered heterogeneous, in BLCA, TMB is acknowleged to be a potential indicator reflecting ICB response efficiency (Chan et al., 2019; Liu et al., 2019). However, only a minor patients received ICB therapy achieved ideal outcomes. Therefore, exploring novel biomarkers for distinguishing specific groups of BLCA patients with an inflamed TME is necessary for increasing the ICB response rates.

IFN-γ signaling has been well-recognized as a critical mediator of tumor cell immunogenicity, which could help promote recognize and eliminate tumor cells (Dighe et al., 1994). IFN-γ expression has been proven to potentially predict clinical outcomes for multiple cancer types (Fridman et al., 2012), and is associated with mortality and disease risk of BLCA as well (Gillezeau et al., 2022). Besides, IFN-γ-induced was reported to up-regulate PD-ECGF/TP and enhance the cytotoxicity of 5-fluorouracil and 5′-deoxy-5-fluorouridine in BLCA (Li et al., 2002). Notably, IFN-γ-induced cytotoxicity has been revealed as a biomarker of resistance in BLCA (Green et al., 2021). However, whether the IFN-γ signaling could be used as an immunotherapy determinant for BLCA has not been fully confirmed.

In this study, an IFN-γ-related signature was developed in BLCA for predicting prognosis, mutation, tumor microenvironment, and immunotherapy. To date, we come first to comprehensively evaluate the predictive values of IFN-γ-related signature in BLCA.

## Materials and methods

### Data collection and procession

The transcriptome data and clinical information of BLCA samples were collected from The Cancer Genome Atlas (TCGA). The FPKM values of the raw matrix were transformed into TPM values for follow-up studies. The TCGA BLCA dataset included 403 BLCA samples and 19 normal samples. Four immunotherapy cohorts were collected, including IMvigor210 (248 samples), Gide (32 samples), Van Allen (42 samples) and Lauss (25 samples). Besides, the copy number variation (CNV) data of BLCA samples in the TCGA BLCA dataset, processed with GISTIC 2.0, were downloaded from the UCSC Xena data portal (http://xena.ucsc.edu/). Drug information, including 184 common anticancer drugs and the corresponding target genes were collected from the DrugBank database (www.drugbank.ca).

### The Xiangya real-world cohort

According to our previous studies, the Xiangya real-world cohort was based on BLCA samples after surgical resections in the Xiangya Hospital, Central South University. The Xinagya real-world cohort, already uploaded with the number as GSE188715, included 57 BLCA samples sequenced by the BGISEQ-500 platform (BGI-Shenzhen, China) (Liu et al., 2021a; Hu et al., 2021). The TPM values of the raw matrix were used for the follow-up analysis.

### Development of the IFN-γ-related signature in TCGA BLCA cohort

IFN-γ related genes have never been systematically summarized as a list. Originally, we collected IFN-γ-related genes from previous studies as comprehensively as possible (Gao et al., 2016; Hu et al., 2020). The least absolute shrinkage and selector operation (LASSO) regression analysis was first performed on these genes for dimension reduction. Each gene can be regarded as a factor, and factors which contribute relatively less to final outcomes of the analysis were assigned the value zero. Remaining factors, genes with non-zero coefficients, were ultimately selected for multivariable Cox regression analysis and used as variables to construct the IFN-γ-related signature. The formula is as follows: Risk score = ∑IFNi*RNAi , where IFNi is the coefficient of the genes and RNAi is the expression value of the genes in multivariable Cox regression analysis. To evaluate the performance of the IFN-γ-related signature, we simultaneously perform the time-dependent receiver operating characteristic (ROC) and calibration curves using the R package “timeROC” in the TCGA BLCA cohort, Xiangya real-world cohort, and IMvigor210 cohort.

### Development of a nomogram

The univariate and multivariate Cox regression analyses were adopted to filtrate independent prognostic factors from clinicopathologic characteristics and the IFN-γ-related signature. After analyzing results, prognostic factors in univariate Cox analysis were screened and integrated to construct a nomogram. The performance of the nomogram was evaluated by the time-dependent ROC and calibration curves using the R package “timeROC” in the TCGA BLCA dataset and the Xiangya real-world cohort.

### The mutation landscape of the IFN-γ-related signature

The mutation landscape of the IFN-γ-related signature was visualized based on CNV data using the R package “maftools.” Somatic mutation data of BLCA samples in the TCGA BLCA dataset was used to calculate the tumor mutation burden (TMB).

### The immunological characteristics of the IFN-γ-related signature

In our previous studies, relevant immunological characteristics and algorithms in the TME were described in detail (Liu et al., 2021a; Hu et al., 2021). The cancer immunity cycle is composed of seven key steps: the release and presentation of cancer cell antigens (Steps 1 and 2), the priming and activation of the immune system (Step 3), then the trafficking and infiltration of immune cells into tumors (Steps 4 and 5), finally recognizing and killing cancer cells by T cells (Steps 6 and 7) (26). The cancer immunity cycle and immune infiltrating cells involved were quantified using the single-sample gene-set enrichment analysis (ssGSEA) in BLCA samples.

### Drug sensitivity and immunotherapy response prediction within the IFN-γ-related signature

We calculated the sensitivity of anticancer drugs using data downloaded from the DrugBank. The predictive value of the IFN-γ-related signature for the immunotherapy was validated in three immunotherapy cohorts, namely the Gide, Van Allen, and Lauss.

### Statistical analysis

Correlation coefficients were determined by the Spearman and distance correlation analyses. Normally distributed continuous variables between the two groups were compared using the *t*-test, while the non-normally ones between the two groups were compared using the Mann-Whitney *U* test instead. Chi-square or Fisher exact tests were used for comparison between categorical variables. The “survcutpoint” function from the R package “survminer” for the maximum rank statistic was used to determine the optimal cutoff value of the IFN-γ-related signature. The survival curves were generated using the Kaplan-Meier method, while the statistical significance was determined using the log-rank test. The threshold of significance was set at *p* < .05, and all statistical tests were two-sided. R project (version 3.6.3, http://www.r-project.org) was used for all analyses.

## Results

### The expression pattern of the IFN-γ-related genes in the TCGA BLCA dataset

**FIGURE 1:**
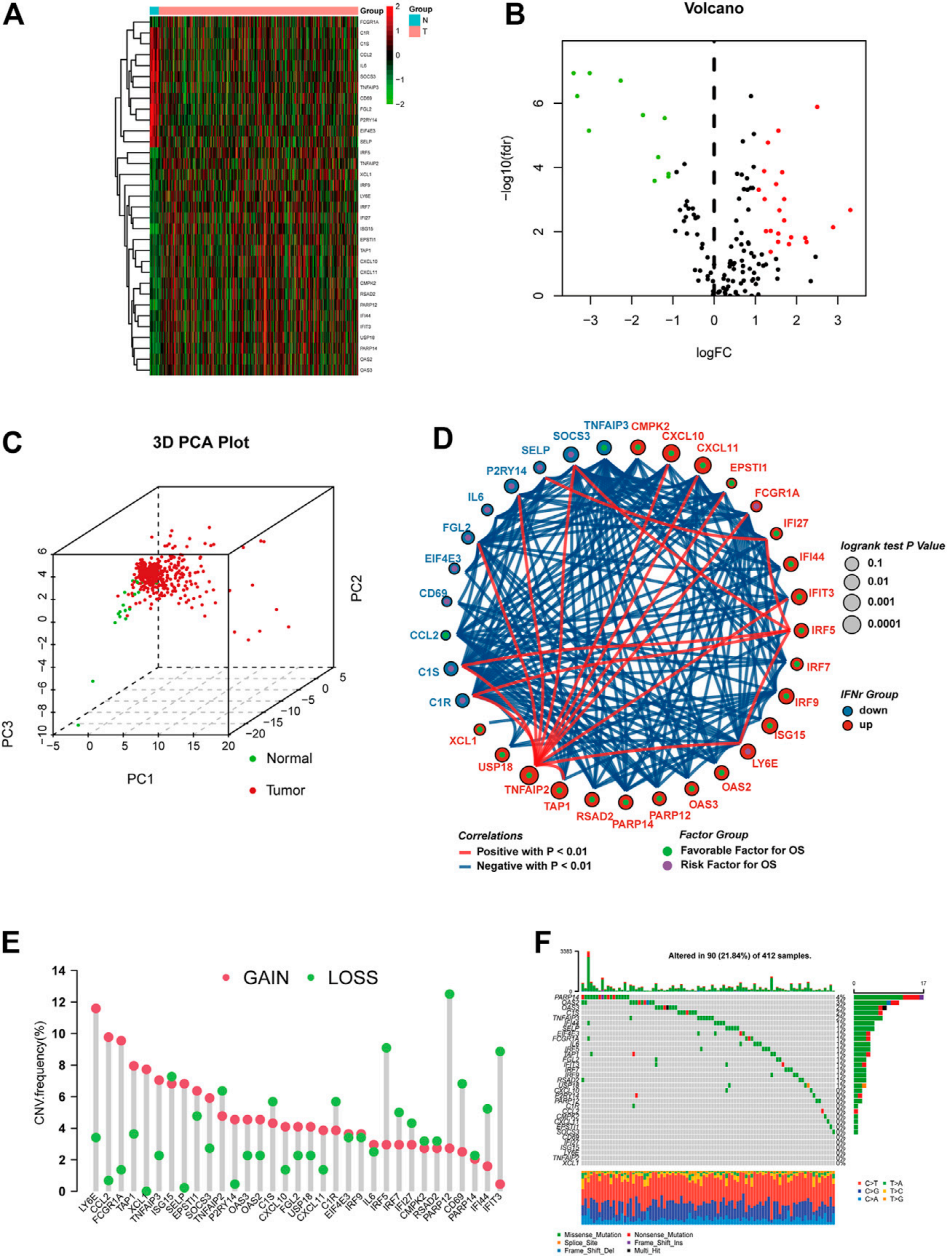
The expression pattern of the IFN-γ-related genes in the TCGA BLCA dataset. **(A)** Heatmap depicting the expression differences of the IFN-γ-related genes between BLCA samples and normal samples. **(B)** Volcano plot for the expression differences of the IFN-γ-related genes between BLCA samples and normal samples. **(C)** 3D PCA plot depicting the expression differences of the IFN-γ-related genes between BLCA samples and normal samples. **(D)** Correlations between the IFN-γ-related genes. The size of the circle, calculated by the log-rank test and ranging from .1 to .0001, represents the prognosis of each gene. Green dots represent favorable factors, while purple dots represent risk factors. The color of the lines represents the correlation between the IFN-γ-related genes. Blue represents a negative correlation, and red represents a positive correlation. **(E)** The CNV frequency of the IFN-γ-related genes. The column represents the count, and the color represents gains or losses. Red represents gains, and blue represents losses. **(F)** The mutation frequency of the IFN-γ-related genes. The upper bar plot represents TMB. The number on the right represents the mutation frequency in each IFN-γ-related gene. The right bar plot represents the proportion of each variant type.

Differential analysis performed on the IFN-γ-related genes between BLCA samples and normal samples was shown in Figures 1A, B. Both sample sets (the BLCA set and normal one) could be clearly separated by IFN-γ-related genes (Figure 1C). Correlations between every two IFN-γ-related genes were shown in Figure 1D, in which 22 IFN-γ-related genes were favorable factors, while the remaining 11 were risk factors. The CNV frequency of IFN-γ-related genes was shown in Figure 1E. Roughly a half of the genes showed CNV loss, while the other half reversed. The mutation frequency of the IFN-γ-related genes was shown in Figure 1F, in which PARP14, OAS2, OAS3, C1S, and TNFAIP3 were the top five mutated IFN-γ-related genes.

### Development of the IFN-γ-related signature in the TCGA BLCA dataset

**FIGURE 2:**
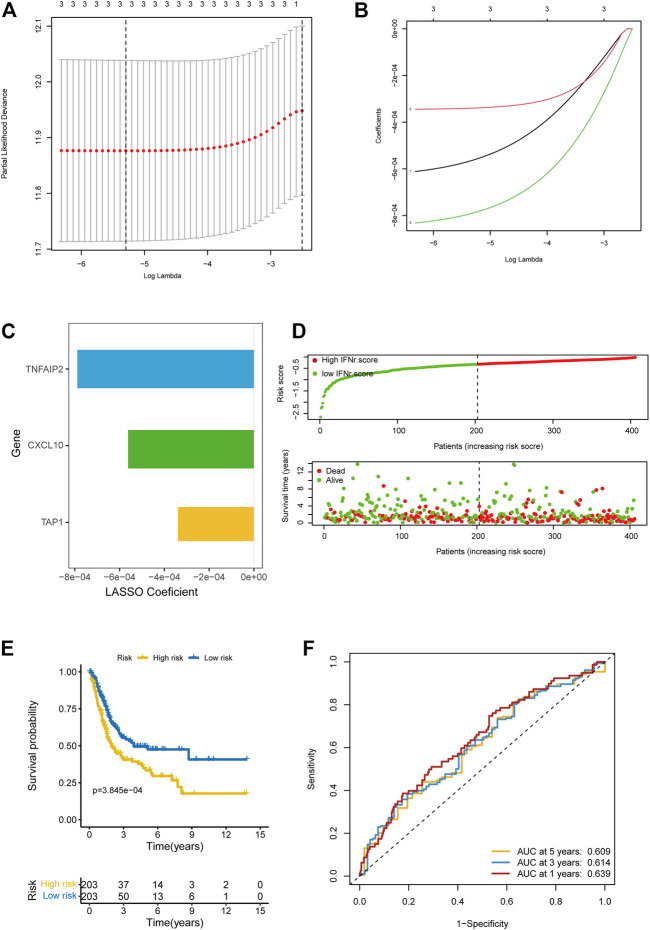
Development of the IFN-γ-related signature in the TCGA BLCA dataset. **(A)** The partial likelihood deviance of the IFN-γ-related genes in LASSO regression analysis. **(B)** The coefficients of the IFN-γ-related genes in LASSO regression analysis. **(C)** The coefficients of the IFN-γ-related genes in LASSO regression analysis. **(D)** The distribution of the IFN-γ-related signature in BLCA samples. **(E)** The survival curves of the two IFN-γ-related signature score groups. **(F)** 1-year, 3-year, and 5-year ROC of the IFN-γ-related signature.

We successfully reduced the dimension of IFN-γ-related genes using LASSO regression analysis (Figure 2A). The coefficients of the IFN-γ-related genes in LASSO regression analysis were shown in Figure 2B. TNFAIP2, CXCL10, and TAP1 were finally included for the multivariable Cox regression analysis. Figure 2C displayed the coefficients of these three genes. The distribution of the IFN-γ-related signature in BLCA samples was shown in Figure 2D. BLCA patients with high IFN-γ-related signature scores were associated with decreased survival time (Figure 2E). The accuracy of the signature in predicting 1-year, 3-year, and 5-year OS was .609, .614, and .639 respectively (Figure 2F).

### Development of a nomogram

**FIGURE 3:**
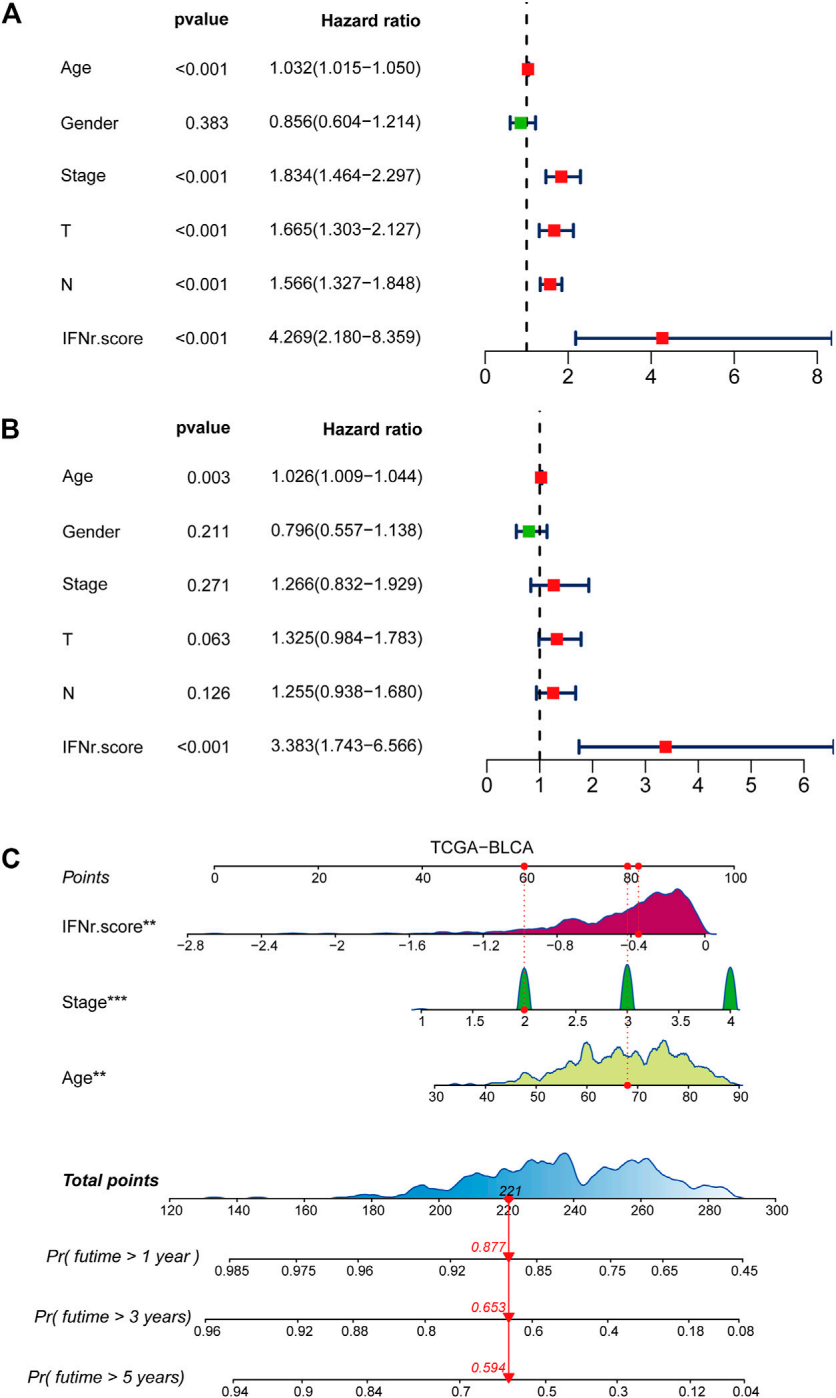
Development of a nomogram. **(A)** Univariate Cox regression analysis on the clinical factors including the IFN-γ-related signature in the TCGA BLCA dataset. **(B)** Multivariate Cox regression analysis on the clinical factors including the IFN-γ-related signature in the TCGA BLCA dataset. **(C)** Nomogram based on clinical factors including the IFN-γ-related signature in the TCGA BLCA dataset.

**FIGURE 4:**
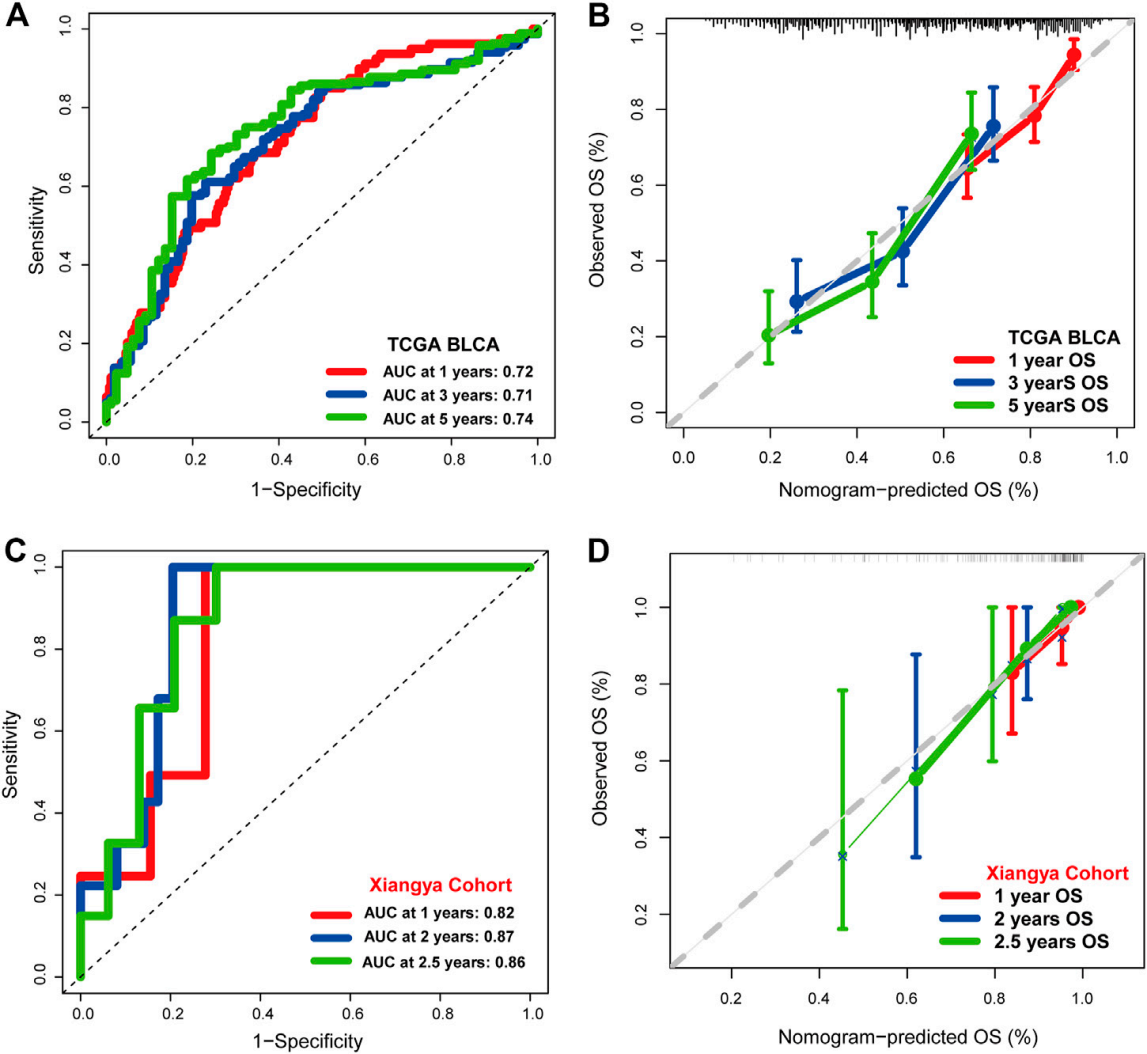
The prognostic value of the nomogram. **(A)** 1-year, 3-year, and 5-year ROC of the nomogram in the TCGA BLCA dataset. **(B)** 1-year, 3-year, and 5-year calibration curves of the nomogram in the TCGA BLCA dataset. **(C)** 1-year, 3-year, and 5-year ROC of the nomogram in the Xiangya real-world cohort. **(D)** 1-year, 3-year, and 5-year calibration curves of the nomogram in the Xiangya real-world cohort.

Univariate Cox regression analysis was performed on clinical characters including the IFN-γ-related signature in the TCGA BLCA cohort. Results revealed that the age, tumor stage, TN grading system, and the IFN-γ-related signature were independent prognostic factors (Figure 3A). Multivariate Cox regression analysis was ultimately performed likewise in the TCGA BLCA dataset, in which age and the IFN-γ-related signature were independent prognostic factors (Figure 3B). Nomogram was constructed based on clinical factors including the IFN-γ-related signature in the TCGA BLCA dataset (Figure 3C).1-year, 3-year, and 5-year ROC of the nomogram in the TCGA BLCA dataset had respective values of .72, .71, and .74 (Figure 4A). 1-year, 3-year, and 5-year calibration curves of the nomogram in the TCGA BLCA dataset were shown in Figure 4B. 1-year, 3-year, and 5-year ROC of the nomogram in the Xiangya real-world cohort had values of .82, .87, and .86 (Figure 4C). 1-year, 3-year, and 5-year calibration curves of the nomogram in the Xiangya real-world cohort were shown in Figure 4D.

### The mutation landscape of the IFN-γ-related signature in the TCGA BLCA dataset

**FIGURE 5:**
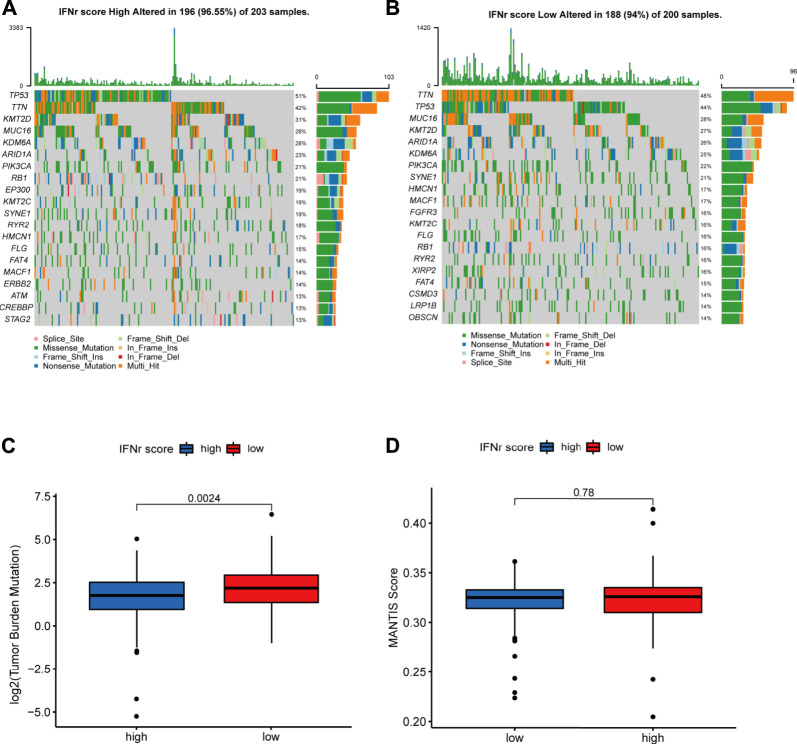
The mutation landscape of the IFN-γ-related signature in the TCGA BLCA dataset. **(A)** The top-ranked mutated genes in the high IFN-γ-related signature score group. **(B)** The top-ranked mutated genes in the low IFN-γ-related signature score group. **(C)** The TMB levels in the two IFN-γ-related signature score groups. **(D)** The MANTIS score in the two IFN-γ-related signature score groups.

TP53, TTN, KMT2D, MUC16, and KDM6A were the top five mutated genes in the high IFN-γ-related signature score group (Figure 5A). TTN, TP53, MUC16, KMT16, and ARID1A were the top five mutated genes in the low IFN-γ-related signature score group (Figure 5B). The high IFN-γ-related signature score group was significantly associated with a lower TMB level (Figure 5C). However, there was no significant correlation between the MANTIS score and the IFN-γ-related signature (Figure 5D).

### The immunological characteristics of the IFN-γ-related signature in the TCGA BLCA dataset

**FIGURE 6:**
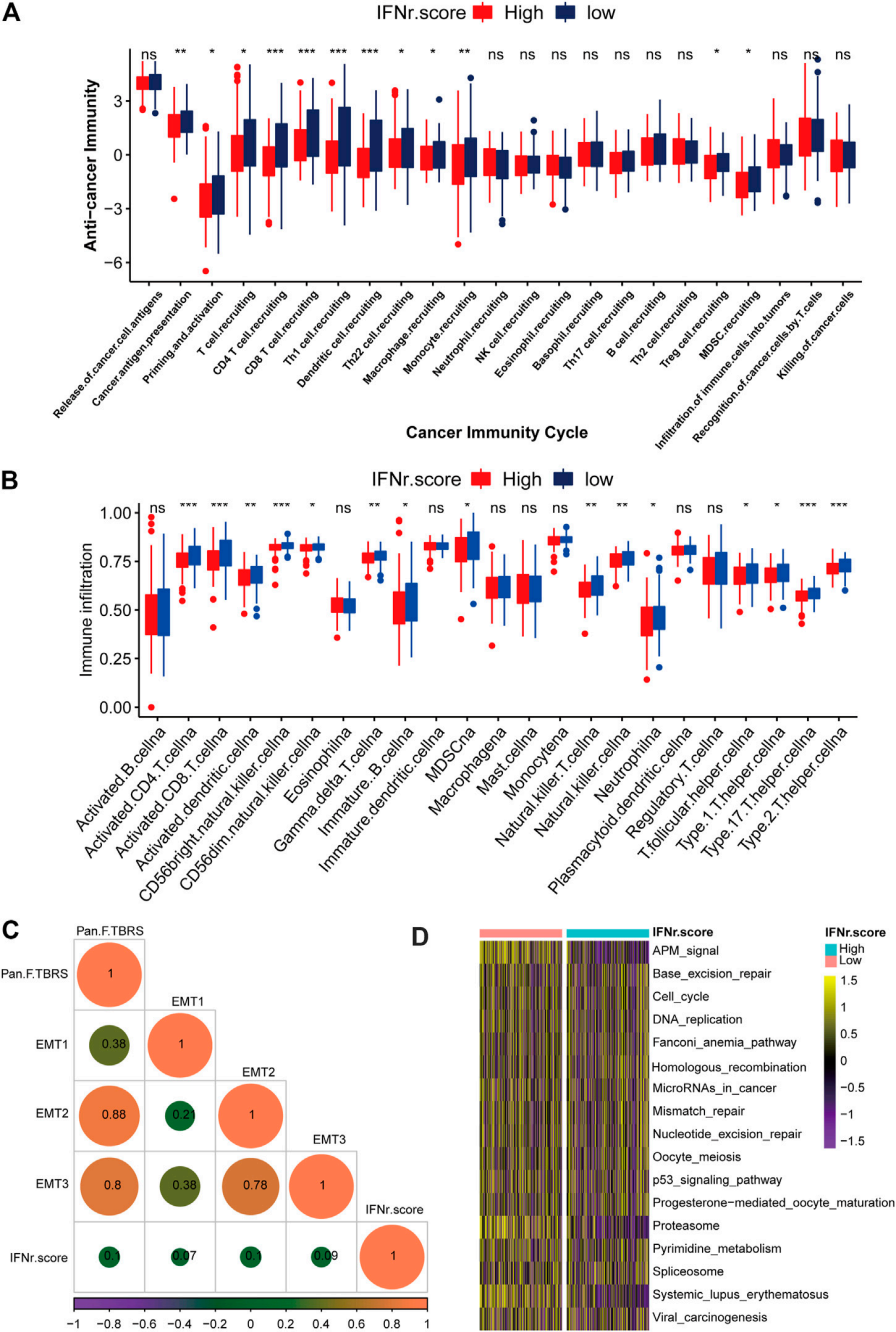
The immunological characteristics of the IFN-γ-related signature in the TCGA BLCA dataset. **(A)** Cancer immunity cycles between the two IFN-γ-related signature score groups. **(B)** Immune infiltrating cells between the two IFN-γ-related signature score groups. **(C)** Correlations between the IFN-γ-related signature and stroma-activated pathways. **(D)** Immunotherapy-predicted pathways between the two IFN-γ-related signature score groups. The left bar represents log10 *p*-values, the red bar represents activated pathways, and the blue bar represents inhibited pathways. (ns, Not Significant; *p < .05; **p < .01; ***p < .001; ****p < .0001).

As was shown in Figure 6A, low IFN-γ-related signature scores significantly indicated some cancer immunity cycles including T cell recruiting, Th 1 cell recruiting, and macrophage recruiting. Besides, the low IFN-γ-related signature scores were generally associated with immune infiltrating cells including activated CD4 cells, activated CD8 cells, and natural killer T cells (Figure 6B). Correlations between the IFN-γ-related signature and each stroma-activated pathway were shown in Figure 6C. Immunotherapy-predicted pathways were relatively more active in the low IFN-γ-related signature score group (Figure 6D). We divided TCGA samples into different binary groups according to the sex and stage. Results of validating our IFN-γ signature in female and male groups, high stage and low stage groups proved the conclusion as expected (Supplementary Figure S1–S4).

### The immunological characteristics of the IFN-γ-related signature in the Xiangya real-world cohort

**FIGURE 7:**
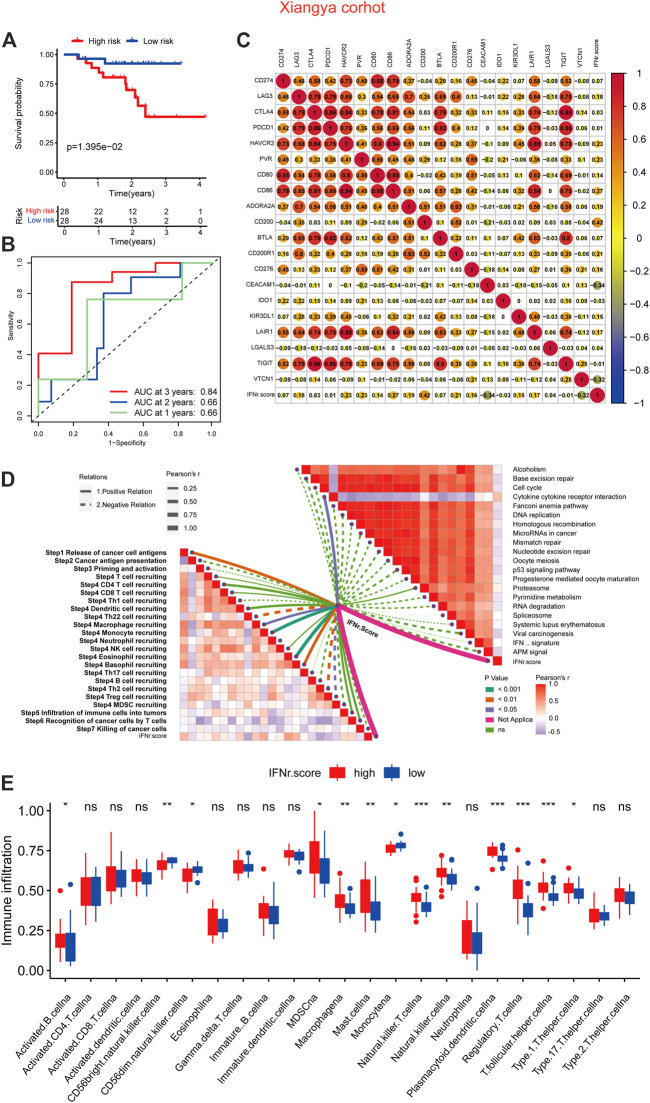
The immunological characteristics of the IFN-γ-related signature in the Xiangya real-world cohort. **(A)** The survival curves of the two IFN-γ-related signature score groups. **(B)** 1-year, 3-year, and 5-year ROC of the IFN-γ-related signature. **(C)** Correlations between the IFN-γ-related signature and immune checkpoint molecules. **(D)** Correlations between the IFN-γ-related signature and cancer immunity cycles and immunotherapy-predicted pathways. **(E)** Correlations between the IFN-γ-related signature and immune infiltrating cells.

BLCA patients with a high IFN-γ-related signature score were associated with shorter survival time (Figure 7A). 1-year, 3-year, and 5-year ROC of the IFN-γ-related signature had values of .84, .66, and .66, respectively (Figure 7B). The signature also showed negative correlation with multiple immune checkpoint molecules, including CD274, LAG3, CTLA4, PDCD1, and HAVCR2 (Figure 7C). As expected, the signature was negatively associated with cancer immunity cycles, including T cell recruiting, Th 1 cell recruiting, and macrophage recruiting (Figure 7D). The IFN-γ-related signature was negatively associated with immunotherapy-predicted pathways, including APM signal, microRNAs in cancer, mismatch repair, cell cycle, and p53 signaling pathway (Figure 7D). In addition, the low signature score group was generally significantly associated with immune infiltrating cells, including activated CD4 cells, activated CD8 cells, and natural killer T cells (Figure 7E).

### The immunological characteristics of the IFN-γ-related signature in the IMvigor210 cohort

**FIGURE 8:**
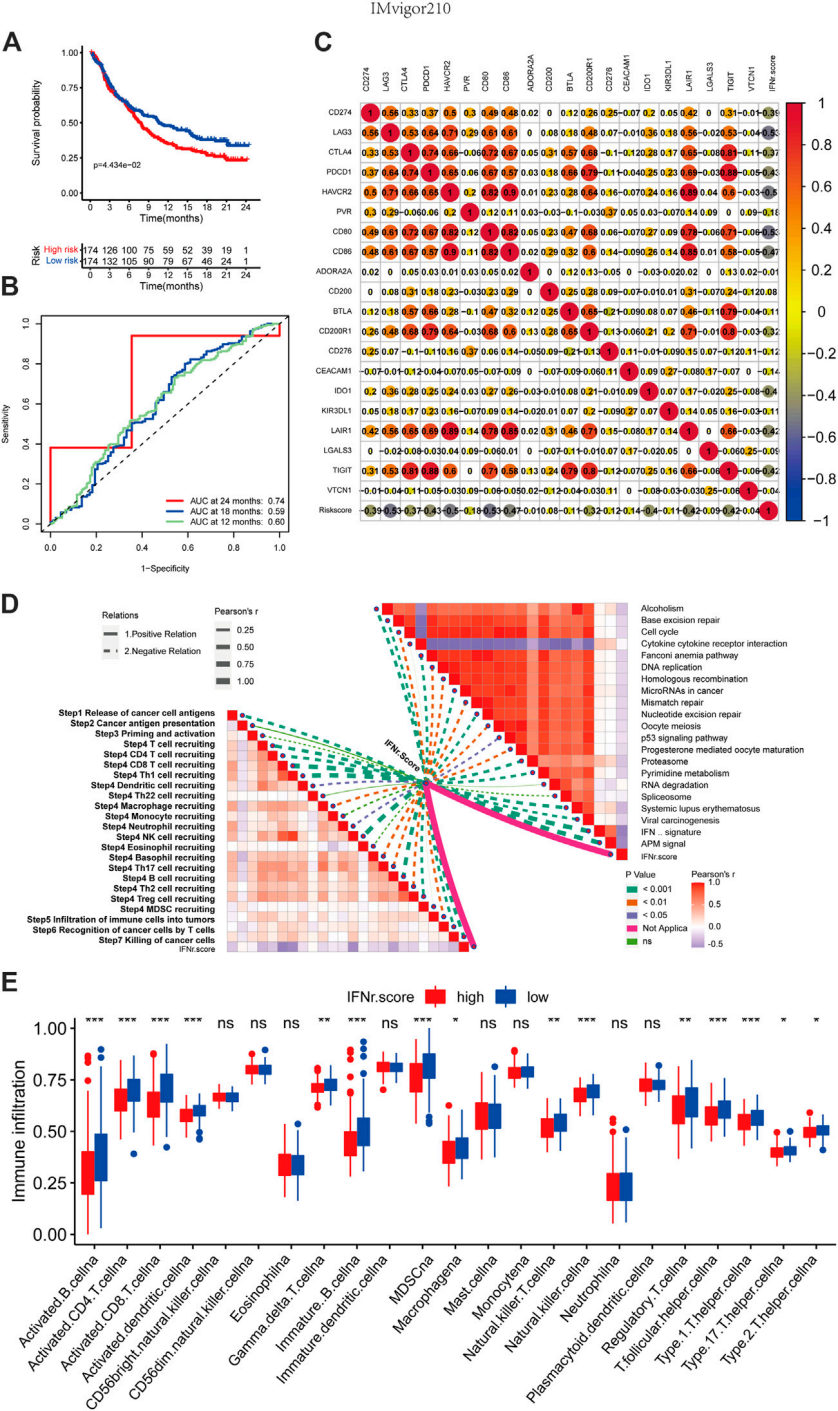
The immunological characteristics of the IFN-γ-related signature in the IMvigor210 cohort. **(A)** The survival curves of the two IFN-γ-related signature score groups. **(B)** 1-year, 3-year, and 5-year ROC of the IFN-γ-related signature. **(C)** Correlations between the IFN-γ-related signature and immune checkpoint molecules. **(D)** Correlations between the IFN-γ-related signature and cancer immunity cycles and immunotherapy-predicted pathways. **(E)** Correlations between the IFN-γ-related signature and immune infiltrating cells.

BLCA patients with high IFN-γ-related signature scores were associated with decreased survival time (Figure 8A). 1-year, 3-year, and 5-year ROC of the IFN-γ-related signature had values of .74, .59, and .6 (Figure 8B). The IFN-γ-related signature was negatively associated with multiple immune checkpoint molecules, including CD274, LAG3, CTLA4, PDCD1, and HAVCR2 (Figure 8C). The IFN-γ-related signature was negatively associated with cancer immunity cycles, including T cell recruiting, Th 1 cell recruiting, and macrophage recruiting (Figure 8D). The IFN-γ-related signature was negatively associated with immunotherapy-predicted pathways, including APM signal, microRNAs in cancer, mismatch repair, cell cycle, and p53 signaling pathway (Figure 8D). In addition, the low IFN-γ-related signature scores were generally significantly associated with immune infiltrating cells, including activated CD4 cells, activated CD8 cells, and natural killer T cells (Figure 8E).

### Drug prediction and immunotherapy response prediction of the IFN-γ-related signature

**FIGURE 9:**
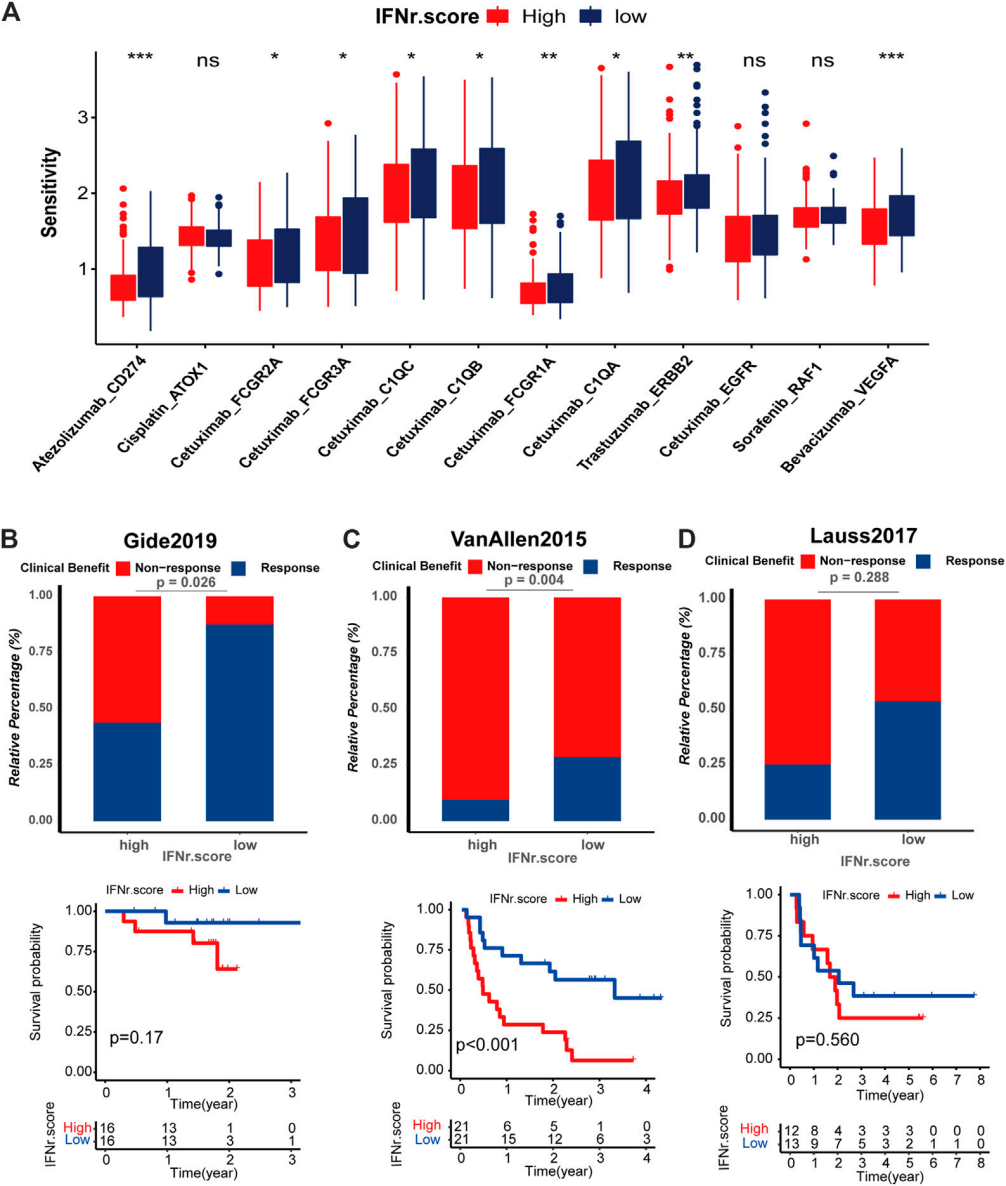
Drug prediction and immunotherapy response prediction of the IFN-γ-related signature. **(A)** The sensitivity of the anticancer drugs in the two IFN-γ-related signature score groups. The predictive value of the IFN-γ-related signature for the immunotherapy was validated in three immunotherapy cohorts, including. **(B–D)** Response of melanoma patients with high and low IFN-γ-related signature scores to ICB in Gide, Van Allen and Lauss respectively.

We predicted the sensitivity of BLCA patients to common drugs. Atezolizumab_CD274, Cetuximab_FCGR2A, Cetuximab_FCGR3A, Cetuximab_C1QC, Cetuximab_C1QB, Cetuximab_FCGR1A, Cetuximab_G1QA, Trastuzumab_ERBB2, and Bevacizumab_VEGFA had significantly lower drug sensitivity in the high IFN-γ-related signature score group (Figure 9A). Likewise, melanoma patients with high IFN-γ-related signature scores were less likely to respond to ICB (Figures 9B–D). Notably, melanoma patients with high IFN-γ-related signature scores were associated with decreased survival time (Figure 9C).

## Discussion

Increasing evidence constantly confirmed that IFN-γ plays a critical role in the tumorigenicity and immunogenicity of various cancers (Jorgovanovic et al., 2020; Stifter et al., 2020). Specially, IFN-γ is a cytokine that physiologically promotes innate and adaptive immune responses, while preventing the development of primary and transplanted tumors (Burke and Young, 2019). However, the potential roles of IFN-γ in the prognosis and especially in the TME of BLCA remain unclear. Recently, mining markers precisely predicting prognosis and survival in cancers based on large-scale bioinformatic analysis has received much more attention in the big data era than ever before. Several markers have been proven robust in predicting survival outcomes and immunotherapy responses (Zhang et al., 2022a; Zhang et al., 2022b). Thus, we aimed to explore the predictive value of IFN-γ and its related genes in BLCA using comprehensive bioinformatics on internal datasets and external real-world validation cohort.

Our study originally proved that most IFN-γ-related genes were risk factors in BLCA.

After summarizing IFN-γ-related genes, a corresponding signature was successfully developed in TCGA BLCA cohort. The predictive signature consisted of three genes, namely TNFAIP2, CXCL10, and TAP1. From with, TNFAIP2, a primary response gene of TNFα, is highly expressed in immune cells and the urinary bladder cells (Jia et al., 2016; Niwa et al., 2019). TNFAIP2 has been reported to promote proliferation, angiogenesis, migration, and invasion of cancer (Jia et al., 2018). CXCL10, CXCL9, CXCL11/CXCR3 is an important axis for immune activation, which is necessary for developing novel cancer therapy (Tokunaga et al., 2018). In addition, a significantly negative correlation between TAP1 and survival in breast, lung, liver, and ovarian cancer is revealed (Tabassum et al., 2021).

The IFN-γ-related signature could predict the survival outcomes of BLCA patients in the TCGA BLCA dataset, Xiangya real-world cohort and IMvigor210 cohort. Cox regression analysis determined that the signature was an independent prognostic factor as age, tumor stage, and TN grading system in the predictive nomogram. The constructed nomogram performs robustly in predicting the survival outcomes of BLCA patients. As generally agreed, the TNM staging system is the most widely accepted and most commonly used system for BLCA (Adsay et al., 2012). The IFN-γ-related signature showed sensationally superior performance compared to the TNM staging system regarding predicting the prognosis of BLCA patients. TP53 is an important tumor suppressor gene that is frequently mutated in cancer (Donehower et al., 2019). While MUC16 and TTN genes mutation were previously reported to correlate with prognosis, tumor mutation burden, and immunotherapy efficacy in cancers (Yang et al., 2020). KMT2D deficiency was found to impair super-enhancers to confer a glycolytic vulnerability in lung cancer (Alam et al., 2020). KDM6A-ARHGDIB axis could block metastasis of BLCA by inhibiting Rac1 (Liu et al., 2021b). In accordance with these findings, TP53, TTN, KMT2D, MUC16, and KDM6A were the top five mutated genes in the high IFN-γ-related signature score group.

The core part of immunotherapy is to help the immune system recognize and destroy tumor cells through enhancing the reaction of immune cells to present tumor antigens (Frankel et al., 2017). TME, composed of cancer cells, non-cancerous cells (mainly immune infiltrating cells), and secreted cytokines, has emerged as a promising mediator for immunotherapy (Bejarano et al., 2021). An immune hot TME, which is infiltrated by more immune cells, is more likely to present a better ICB response (Wang et al., 2022). The IFN-γ-related signature was negatively associated with cancer immunity cycles, immunotherapy-predicted pathways, and immune infiltrating cells in the TCGA BLCA dataset, Xiangya real-world cohort, and IMvigor210 cohort. As the most important determinant for ICB, immune checkpoint molecules have been widely studied in the past few decades. CD274, LAG3, CTLA4, PDCD1, and HAVCR2 have been the most promising immune checkpoint molecules with satisfying results in clinical trials (Wang et al., 2022). As expected, the IFN-γ-related signature was negatively associated with multiple immune checkpoint molecules, including CD274, LAG3, CTLA4, PDCD1, and HAVCR2. Besides, the high IFN-γ-related signature score group was significantly associated with a lower TMB level. These results indicate potential lower response rates in BLCA patients with high IFN-γ-related signature scores. The direct immunotherapy response prediction of the IFN-γ-related signature in Gide, Van Allen, and Lauss cohorts proved this finding. Furthermore, IFN-γ-related signature could predict drug sensitivity of Atezolizumab_CD274, Cetuximab_FCGR2A, Cetuximab_FCGR3A, Cetuximab_C1QC, Cetuximab_C1QB, Cetuximab_FCGR1A, Cetuximab_G1QA, Trastuzumab_ERBB2, and Bevacizumab_VEGFA.

To sum up, an IFN-γ-related signature was developed in BLCA for predicting prognosis, mutation, tumor microenvironment, and immunotherapy. The potential clinical application of the IFN-γ-related signature is expected to be further validated by more clinical cohorts.

## Data Availability

The datasets presented in this study can be found in online repositories. The names of the repository/repositories and accession number(s) can be found in the article/Supplementary Material.

## Supplementary material

SUPPLEMENTARY FIGURE S1 Exploring correlations between the IFN-γ score and TME characteristics in female group. **(A)** Differences in cancer immune cycle activity between high-risk and low-risk groups. **(B)** Correlation between the score and the infiltration of different immune cells. **(C)** Relationship between the TIS and the IFN-γ score. **(D)** Correlation between the IFN-γ score and various common ICB response pathways. **(E)** Correlation between the IFN-γ score and immune checkpoints.

SUPPLEMENTARY FIGURE S2 Exploring correlations between the IFN-γ score and TME characteristics in male group.

SUPPLEMENTARY FIGURE S3 Exploring correlations between the IFN-γ score and TME characteristics in high stage group.

SUPPLEMENTARY FIGURE S4 Exploring correlations between the IFN-γ score and TME characteristics in low stage group.
