# Supplementary figures and images for: An IFN-γ-related signature predicts prognosis and immunotherapy response in bladder cancer: Results from real-world cohorts
Source: Front Genet. 2023 Jan 4;13:1100317. doi: 10.3389/fgene.2022.1100317 (PMC9846040; doi:10.3389/fgene.2022.1100317)

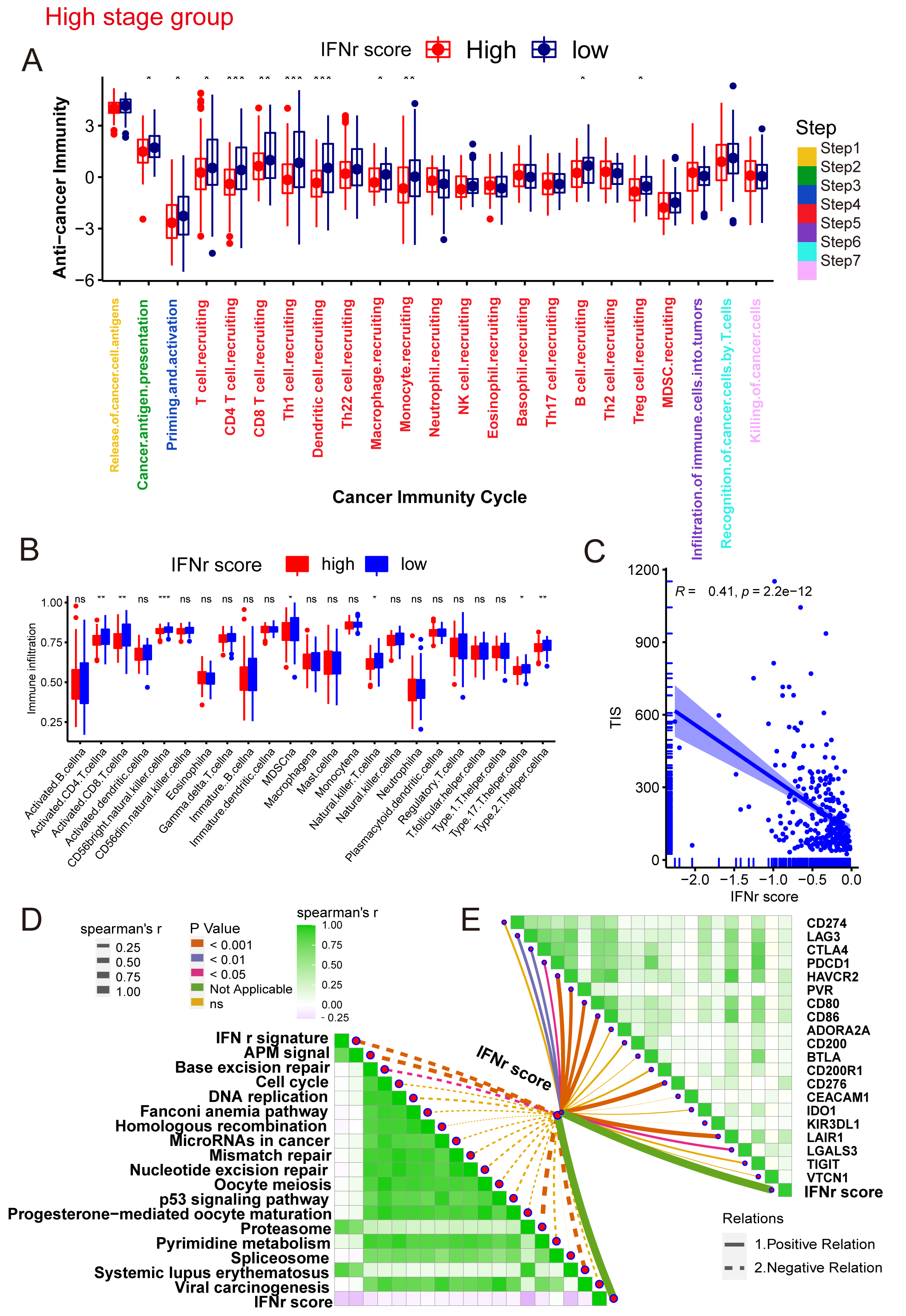

Supplement: Supplementary file 1 [file Image3.tif]

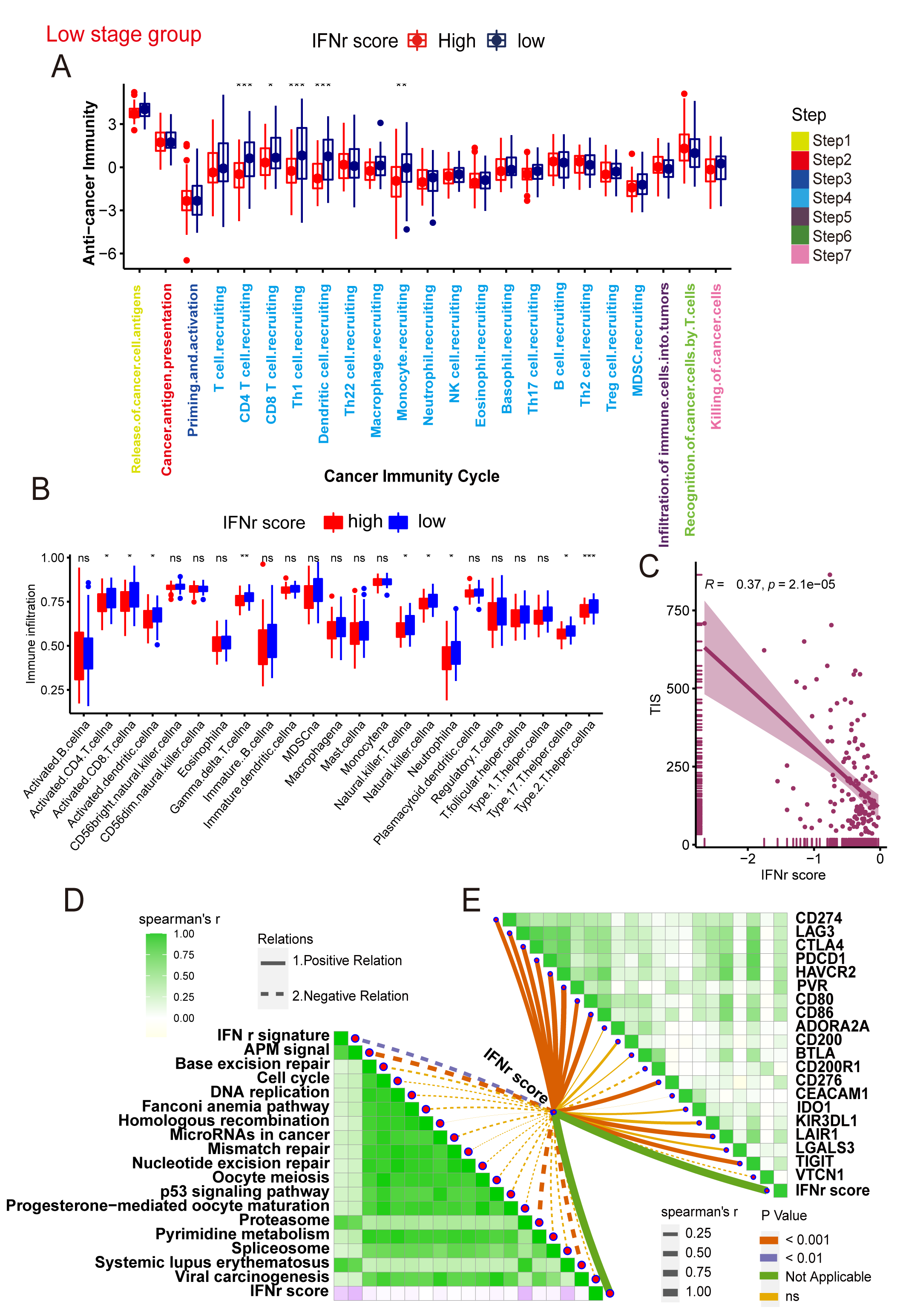

Supplement: Supplementary file 2 [file Image4.tif]

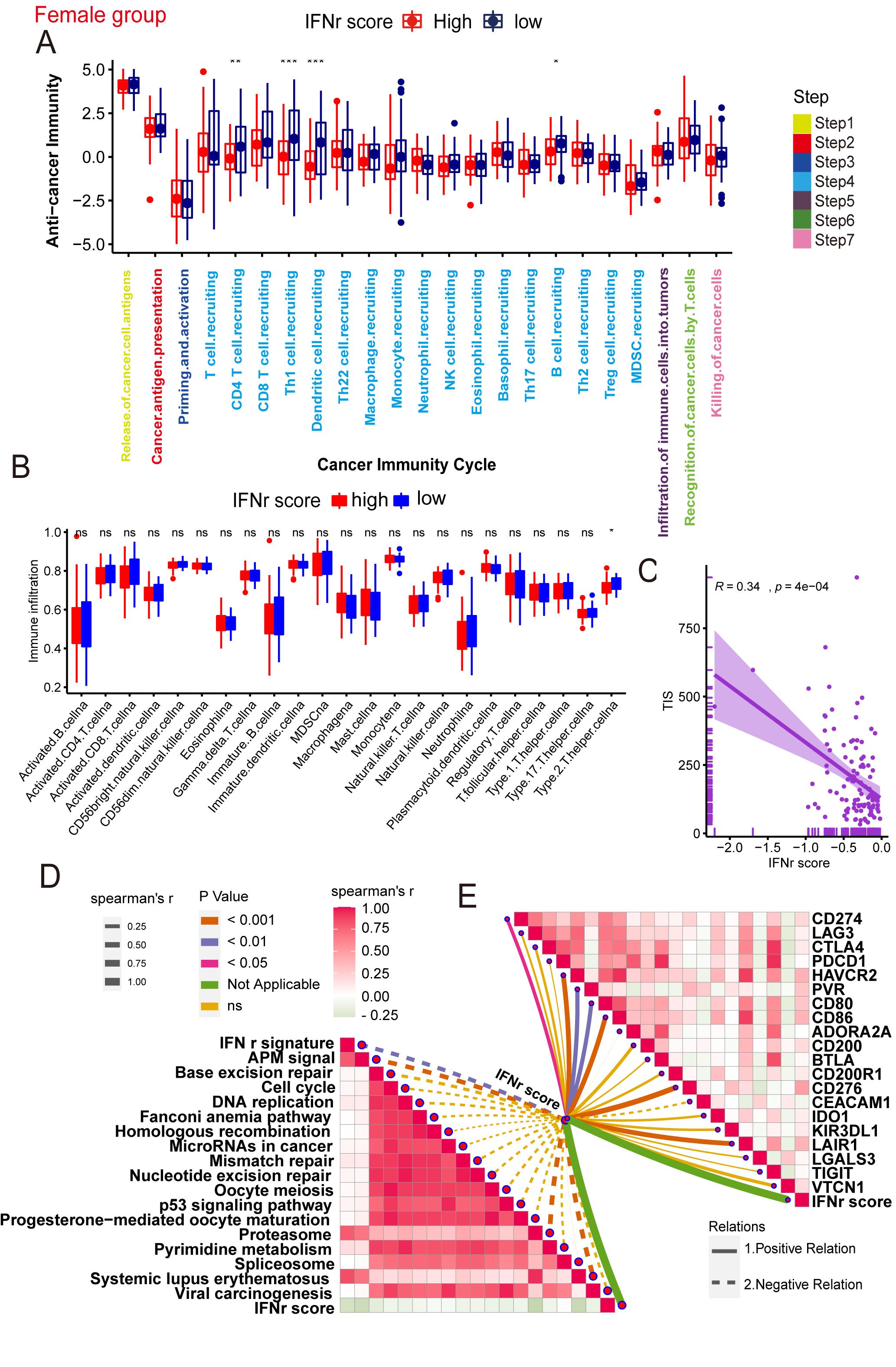

Supplement: Supplementary file 3 [file Image1.jpeg]

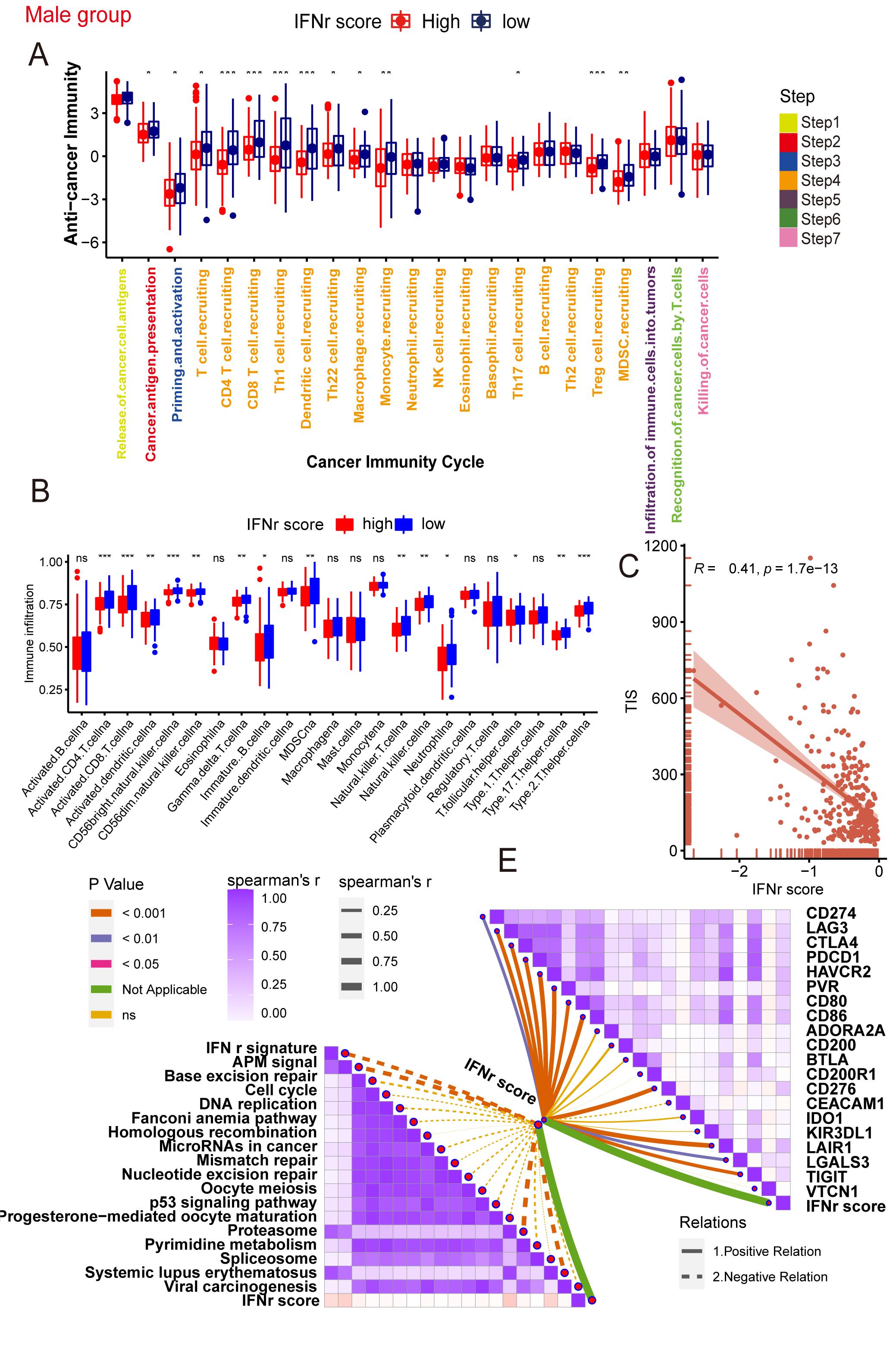

Supplement: Supplementary file 5 [file Image2.jpeg]
